# Effect of dietary zinc source, zinc concentration, and exogenous phytase on intestinal phytate degradation products, bone mineralization, and zinc status of broiler chickens

**DOI:** 10.1016/j.psj.2023.103160

**Published:** 2023-10-02

**Authors:** Hanna Philippi, Vera Sommerfeld, Oluyinka A. Olukosi, Wilhelm Windisch, Alessandra Monteiro, Markus Rodehutscord

**Affiliations:** ⁎ Institute of Animal Science, University of Hohenheim, Stuttgart 70599, Germany; † Department of Poultry Science, University of Georgia, Athens, GA 30602, USA; ‡ Chair of Animal Nutrition, TUM School of Life Sciences, Technical University of Munich, Freising 85354, Germany; # Animine, Annecy 74960, France

**Keywords:** phytate degradation, zinc, phytase, bone mineralization, broiler

## Abstract

This study aimed to determine the effect of Zn source and dietary level on intestinal *myo*-inositol hexakisphosphate (**InsP_6_**) disappearance, intestinal accumulation of lower InsP and *myo*-inositol (**MI**), prececal mineral digestibility, bone mineralization, and Zn status of broilers without and with exogenous phytase in the feed. Male Ross 308 broilers were allocated in groups of 10 to 8 treatments with 8 pens each. Experimental diets were fed from d 7 to d 28 and contained 33 mg/kg dry matter plant-intrinsic Zn. Experimental factors were phytase supplementation (0 or 750 FTU/kg) and Zn source (none [0 mg/kg Zn], Zn-sulfate [30 mg/kg Zn], Zn-oxide [30 mg/kg Zn]). Additional treatments with 90 mg/kg Zn as Zn-sulfate or Zn-oxide and phytase were included to test the effect of Zn level. No Zn source or Zn level effects were observed for ADG, feed conversion ratio, prececal P digestibility, intestinal InsP_6_ disappearance, and bone ash concentration. However, those measurements were increased by exogenous phytase (*P* < 0.001), except the feed conversion ratio, which was decreased (*P* < 0.001). Ileal MI concentrations were affected by phytase × Zn source interaction (*P* < 0.030). Birds receiving exogenous phytase and Zn supplementation had the highest MI concentrations regardless of exogenous Zn source, whereas MI concentrations were intermediate for birds receiving exogenous phytase only. Exogenous phytase and exogenous Zn source increased the Zn concentration in bone and blood of broilers (*P* < 0.001). In conclusion, measures of exogenous phytase efficacy were not affected by phytase × Zn source interaction. Further studies are needed to rule out an effect from Zn sources other than those tested in this study and to investigate the effect of Zn supplementation on endogenous phosphatases. The missing effect of increasing Zn supplementation from 30 to 90 mg/kg in phytase-supplemented diets gives reason to reconsider the Zn supplementation level used by the industry.

## INTRODUCTION

Zinc is an essential mineral in all living organisms, as it functions as a cofactor for many enzymes and as a second messenger (Kimura and Kambe, 2016; Brugger and Windisch, 2019). When the Zn supply is insufficient, growth and bone development can be impaired (Zeigler et al., 1961). The Zn supply recommendation for broiler chickens is 50 mg/kg DM according to the Gesellschaft für Ernährungsphysiologie (1999) or 40 mg/kg according to the National Research Council (1994). The intrinsic Zn concentration of common feedstuffs for poultry does not cover the Zn requirement, and the availability of intrinsic Zn is low due to dietary antagonists such as phytate (Maddiaiah et al., 1964). Hence, commercial broiler diets are usually supplemented with Zn, including high safety margins that are of concern in regard to environmental effects.

Phytate is the salt form of phytic acid (*myo*-inositol 1,2,3,4,5,6-hexakisphosphate [**InsP_6_**]) and the primary storage form of P in plant-based feedstuffs. The availability of phytate-P is limited for nonruminant animals and different between animal species (Rodehutscord et al., 2022). Phytases (*myo*-inositol hexaphosphate phosphohydrolases) and other phosphatases are needed to induce cleavage of the phosphate groups from the *myo*-inositol ring, making the phosphate absorbable. In order to increase the digestibility of phytate-P, poultry diets are commonly supplemented with exogenous phytases whose main site of action is the proximal gastrointestinal tract.

Phytate can form insoluble complexes with (divalent) cations, such as Ca and Zn, especially at the pH prevailing in the small intestine (Oberleas et al., 1966; Champagne and Hinojosa, 1987). Calcium, for instance, has been shown to negatively impact InsP_6_ disappearance in the small intestine of broilers at high dietary levels, especially in the presence of high dietary P levels (Sommerfeld et al., 2018b). As the stability of complexes between phytate and Zn was ranked the highest among all cations tested (Maenz et al., 1999), it is crucial to also consider Zn as a potential influencing factor of phytase efficacy. In vitro studies have shown an adverse effect of Zn on phytase efficacy. When Zn was added to a Na-phytate solution, phosphate release by phytase was reduced, with the degree of reduction depending, among others, on the Zn source (Santos et al., 2015). It was hypothesized that the reduction might be due to the formation of insoluble Zn-phytate-complexes (Oberleas et al., 1966) rendering the InsP_6_ unavailable for degradation by phytase. This hypothesis was supported by a study by Augspurger et al. (2004), in which tibia ash was significantly reduced in phytase supplemented diets when Zn was added at a pharmacologic level (800 mg/kg). Besides the Zn level, the solubility of Zn sources could also affect the interaction with phytate, with faster dissolving Zn sources having greater potential for interaction than slower dissolving sources (Cardoso et al., 2021a). This was shown in vitro for 2 Cu sources differing in solubility, where phosphate release by phytase was lower when Cu was supplemented as Cu sulfate compared to Cu oxide (Hamdi et al., 2018). Because Zn-sulfate and Zn-oxide differ in their dissolution speed (Cardoso et al., 2021b), those 2 sources were chosen in the present study. To our knowledge, this hypothesis has never been tested in vivo for nonpharmacologic Zn levels in broilers.

Therefore, the first objective of this study was to investigate whether the supplementation of Zn, depending on the Zn source, affects phytase efficacy by determining intestinal InsP_6_ disappearance, accumulation of lower InsP_x_, P digestibility, and bone mineralization. The second objective was to determine the Zn status, as determined by the Zn concentration of bone, blood, and liver of the birds, and whether it is affected by exogenous phytase, Zn source, and dietary Zn level. It was hypothesized that 1) Zn supplementation reduces phytase efficacy at a dose of 750 FTU/kg; and 2) phytase increases Zn concentration in bone, blood, and liver of birds.

## MATERIAL AND METHODS

### Birds and Housing

The experiment was carried out at the Agricultural Experiment Station of the University of Hohenheim following the German Animal Welfare Legislation and approved by the Regierungspräsidium Tübingen (Approval no. HOH 65/21_460a). A total of 640 male Ross 308 broilers were obtained from a commercial hatchery (Brüterei Süd GmbH & Co. KG, Regenstauf, Germany) and distributed into 64 floor pens (115 × 230 × 260 cm) with wood shavings in groups of 10 birds. Each of the 8 dietary treatments was randomly assigned 8 pens in a randomized complete block design. From d 0 to d 7, birds received a pelleted nutrient-adequate corn–soybean meal-based pre-experimental starter diet according to Gesellschaft für Ernährungs-physiologie (1999). Birds were reallocated on d 7 to ensure even weight distribution across all treatments and blocks and received pelleted experimental grower diets from d 7 to d 28. Feed and tap water were provided ad libitum throughout the experiment. Birds were kept on perforated floors from d 16 to the end to prevent intake of marker (titanium dioxide) from the feces. For the first 3 d, the light schedule was 24L:0D; from d 3 until the end of the experiment, the light schedule was 18L:6D. The room temperature was continuously lowered from 34°C at the beginning to 22°C by the end of the experiment. Birds were checked twice daily to monitor the health status.

### Experimental Diets

Diets were calculated to contain adequate levels of all nutrients according to Gesellschaft für Ernährungsphysiologie (1999), except non-phytate P and Zn. Diets were based on corn, soybean meal, rapeseed meal, and rice bran (Table 1). Titanium dioxide was used as an indigestible marker with an inclusion level of 5 g/kg. Eight experimental diets were prepared in a 2 × 3 + 2 factorial arrangement of treatments. The factors phytase level (0 (**PHY–**) and 750 FTU/kg (**PHY+)**, Quantum Blue, AB Vista, Marlborough, United Kingdom (*Escherichia coli* derived 6-phytase)) and Zn source (none (**Zn–**) or 30 mg/kg of Zn sulfate heptahydrate (**ZnSO_4_+**), Sigma Aldrich, St. Louis, MO or 30 mg/kg of a modified Zn oxide (**ZnO+**), HiZox, Animine, Annecy, France) were included in the full-factorial design. The 2 other treatments were added to test the effect of Zn supplementation level (ZnSO_4_ or ZnO at 90 mg/kg Zn [**Zn++**]) and included exogenous phytase (750 FTU/kg). The lower Zn supplementation level was chosen to meet the bird's requirement of 50 mg/kg DM (Gesellschaft für Ernährungsphysiologie, 1999). The higher Zn level was chosen on the basis of European regulations. For poultry, the Zn content in complete feed, including the intrinsic Zn content, must not exceed 120 mg/kg (DVO (EU) Nr. 2016/1095). The level of exogenous phytase (750 FTU/kg) was chosen in combination with the calculation of a high phytate-P level (3.5 g/kg DM) to ensure sufficient undegraded phytate is present in the small intestine to challenge interactions of phytate and Zn. Experimental diets were prepared by mixing all nonvariable ingredients (Table 1) to obtain a basal diet. The basal diet was then divided into 5 parts and supplemented with the corresponding quantity of ZnSO_4_ (analyzed Zn concentration: 326 g/kg DM) or ZnO (analyzed Zn concentration: 731 g/kg DM) at the expense of corn. The mixtures with 90 mg/kg Zn were supplemented with the exogenous phytase product. The mixtures with 0 and 30 mg/kg Zn were split again and one half of each was supplemented with the exogenous phytase. After mixing, experimental diets were pelleted through a 3-mm pelleting matrix. The temperature of the pellets was recorded immediately after release from the press and did not exceed 78°C. Representative diet samples were taken and ground to pass a 0.5-mm sieve (Retsch ZM200, Retsch GmbH, Haan, Germany) or pulverized by a vibrating cup mill (PULVERISETTE 9, Fritsch GmbH, Idar-Oberstein, Germany).

**Table 1:** Ingredient composition of the basal diets.

Ingredient, g/kg	Pre-experimental	Experimental
Corn	563	553.3
Soybean meal	356	240
Rapeseed meal	0	95
Rice bran	0	45
Soybean oil	35	30
Limestone	13.5	13
Monocalcium phosphate	21	6.5
L-lysine sulfate	0.8	1.2
DL-methionine	3.5	3.0
Titanium dioxide	0	5.0
Sodium chloride	2.1	2.5
Sodium bicarbonate	2.5	3.0
Mineral premix (Zn-free)^1^	0.5	0.5
Vitamin premix^2^	2.0	2.0
Zinc sulfate^3^	0.1	0

1 Mineral premix (BASU Mineralfutter GmbH, Germany) provided per kg of complete diet: 45 mg Ca from calcium carbonate, 74 mg Mn from manganese sulfate, 23 mg Fe from iron sulfate, 7 mg Cu from copper sulfate, 0.6 mg I from calcium iodate, 0.2 mg Se from sodium selenite.

2 Vitamin premix (GELAMIN, Germany), provided per kg of complete diet: 10,000 IU vitamin A, 1,000 IU vitamin D3, 2.4 mg vitamin K3, 0.1 mg biotin, 1 mg folic acid, 3 mg vitamin B1, 6 mg vitamin B2, 6 mg vitamin B6, 30 μg vitamin B12, 50 mg nicotinic acid, 14 mg pantothenic acid.

3 ZnSO_4_ × 7 H_2_O (Sigma Aldrich, MO) was added to the pre-experimental diet to achieve a total Zn concentration of 50 mg/kg DM.

### Experimental Procedures

Birds and feed were weighed on d 7 and d 28 to calculate mortality-corrected average daily feed intake (**ADFI**), average daily gain (**ADG**), and feed conversion ratio (**FCR**) on a pen basis. In order to standardize the gut fill prior to sampling, the feed was withdrawn 2 h before slaughter and access to the feed was allowed again 1 h before slaughter. All birds from a pen were stunned with a gas mixture of 35% CO_2_, 35% N_2_, and 30% O_2_ and one randomly selected bird was weighed individually, killed by decapitation, and trunk blood from this bird was collected in Na fluoride treated tubes. The plasma was obtained by immediate centrifugation at 2,000×*g* for 10 min. In order to obtain serum, the blood was collected in a tube without additive and tubes were centrifuged at 2,000×*g* for 10 min after resting at room temperature for 0.5 to 2 h. The liver was removed from the decapitated bird and weighed. All remaining broilers from the same pen were killed by CO_2_ exposure. Digesta from the duodenum and jejunum combined (**duo+jej**) and the distal two thirds of the section between Meckel's diverticulum and 2 cm anterior to the ileo-ceco-colonic junction (herein defined as ileum) were collected by gentle squeezing and pooled on a pen basis. The left tibiotarsus (tibia) and left foot from 2 randomly selected birds per pen were collected. All samples were frozen immediately at −20°C, and digesta and liver were freeze-dried and stored at 4°C until further processing. Freeze-dried digesta were pulverized in a vibrating cup mill.

### Chemical Analyses

Feed samples were analyzed for DM according to the official methods in Germany (Verband Deutscher Landwirtschaftlicher Untersuchungs- und Forschungsanstalten, 2007). Feed samples and digesta were analyzed for Ti according to the modified wet digestion method of Boguhn et al. (2009) followed by inductively coupled plasma optical emission spectrometry measurement, described by Zeller et al. (2015). For mineral analysis (Ca, P, Zn, Fe, Mn, and Cu), digesta and feed samples were digested using wet microwave digestion with nitric acid, followed by inductively coupled plasma optical emission spectrometry measurement (USEPA, 1994).

To determine InsP_3-6_ isomers, the extraction and measurement in feed and digesta were conducted using the method of Zeller et al. (2015) with slight modifications described by Sommerfeld et al. (2018b). Using this methodology, it is not possible to separate the enantiomers. Because of co-elution, it was not possible to distinguish between isomers Ins(1,2,6)P_3_, Ins(1,4,5)P_3_, and Ins(2,4,5)P_3_. Hence, the term InsP_3x_ will be used for these isomers with unknown proportions. *Myo*-inositol (**MI**) in digesta and plasma were measured as described in Sommerfeld et al. (2018a).

Blood serum was analyzed for Zn, inorganic P (**P_i_**), and Ca by IDEXX GmbH (IDEXX BioAnalytics, Kornwestheim, Germany). Zinc in blood serum was measured by optical emission spectrometry using Thermo-Fisher iCAP-Duo 7000. Calcium in blood serum was determined photometrically by color test in Beckman Olympus AU480. Inorganic P in blood serum was measured photometrically by UV-test in Beckman Olympus AU480.

Tibia was thawed and adhering soft tissue, cartilage caps, and fibula bones were removed manually. Feet were detached at the *articulatio intertarsalis* and used completely below that joint, including skin, claws, and tissues. Subsequently, tibiae and feet were rinsed with distilled water, carefully dabbed, and dried for 48 h (tibiae) and 72 h (feet) at 103°C in a convection oven (VL 115, VWR International GmbH, Darmstadt, Germany). After drying, bone samples were cooled in a desiccator and weighed. Ash content was determined at 600°C in a muffle furnace (L 40/11/B170, Nabertherm GmbH, Lilienthal, Germany) for 24 h (tibiae) and 48 h (feet) for individual tibiae and feet. Ashed tibiae and feet were weighed and pulverized. After wet microwave digestion with nitric acid, ground tibiae and feet ash samples were analyzed for Ca, P, and Zn using inductively coupled plasma optical emission spectrometry. The freeze-dried liver was pulverized and analyzed for Zn using the method described before for feed.

Phytase activity in feed samples was determined by AB Vista (Marlborough, United Kingdom) using the ELISA method and subsequent conversion of results to FTU per kilogram.

Difference between calculated and analyzed Zn concentrations did not exceed 5.5% (Table 2). Analyzed P concentrations were up to 11% higher than calculated, but the deviation between different treatments did not exceed 4%. The deviation of calculated and analyzed phytase activity was less than 10%.

**Table 2:** Calculated and analyzed composition of the experimental diets.

			Ca	Total P	InsP_6_-P	Zn^1^	Fe	Cu	Mn	Phytase activity
Treatment			———g/kg DM———			———–mg/kg DM———				FTU/kg
			Calculated composition							

Phytase, FTU/kg	Zn source	Zn level, mg/kg								

0	None	0	9.1	6.7	3.5	33	156	14	108	<50
0	ZnSO_4_	30				67				<50
0	ZnO	30				67				<50
750	None	0				33				750
750	ZnSO_4_	30				67				750
750	ZnO	30				67				750
750	ZnSO_4_	90				135				750
750	ZnO	90				135				750

			Analyzed composition							

0	None	0	9.5	7.3	3.3	33	431	15	118	<50
0	ZnSO_4_	30	9.4	7.2	3.3	64	341	14	121	<50
0	ZnO	30	9.1	7.2	3.3	67	290	14	117	<50
750	None	0	9.4	7.4	3.3	33	303	15	115	698
750	ZnSO_4_	30	9.3	7.2	3.4	64	302	13	115	767
750	ZnO	30	9.5	7.3	3.3	68	302	16	120	763
750	ZnSO_4_	90	9.4	7.3	3.4	136	300	14	121	766
750	ZnO	90	9.9	7.5	3.4	139	316	14	126	785

1 Analyzed concentration of Zn includes intrinsic and supplemented Zn. Calculated metabolizable energy content was 13.2 MJ/kg DM for all experimental diets. Calculated crude protein content was 229 g/kg DM for all experimental diets.

### Calculations and Statistical Analysis

Average daily gain, ADFI and FCR were calculated on a pen basis for the experimental phase (d 7–d 28) and corrected for mortality.

For performance, digesta, and bone, the pen was considered the experimental unit. The bird was considered the experimental unit for blood and liver since data were obtained from individual birds.

The InsP_6_ disappearance and P and Ca digestibility were calculated for digesta of duo+jej and ileum using the following equation:

$$y\left(X\right)=100-\left\{100\times \left(\frac{\text{Ti}\;\text{in}\;\text{feed}}{\text{Ti}\;\text{in}\;\text{digesta}}\times \frac{X\;\text{in}\;\text{digesta}}{X\;\text{in}\;\text{feed}}\right)\right\}$$

Where y(X) is the disappearance or digestibility of X in % and X is the concentration of Ca, P, or InsP_6_ in feed and digesta.

Data were analyzed in a nested 2-factorial analysis of variance using the MIXED procedure of the software package SAS (version 9.4, SAS Institute Inc., Cary, NC). The following model was chosen:

$$y_{\text{ijkl}}=\mu +\alpha_{i}+\beta_{j\left(i\right)}+\gamma_{k\left(i\right)}+{\left(\beta \gamma \right)}_{\text{jk}\left(i\right)}+\delta_{l}+\varepsilon_{\text{ijkl}}$$

Where $y_{\text{ijkl}}$ is the response variable, μ is the overall mean, α represents the fixed effect of *i*th treatment group (i = PHY+ ZnSO_4_++ or PHY+ ZnO++ or others), β represents the fixed effect of *j*th exogenous phytase level within ith treatment group (j = 0 or 750 FTU/kg), γ represents the fixed effect of *k*th Zn source within ith treatment group (k = Zn– or ZnSO_4_ or ZnO), βγ is the corresponding interaction effect of the jth exogenous phytase level and kth Zn source within ith treatment group, δ is the random effect of *l*th block (l = 1–8), and $\varepsilon_{\text{ijkl}}$ is the residual error. The same model was fitted in a second version, where treatments were re-parameterized as an 8-level treatment factor. The model was: $y_{\text{ijkl}}=\mu +\tau_{\text{ijk}}+\delta_{l}+\varepsilon_{\text{ijkl}}$, with $y_{\text{ijkl}}$ = response variable, μ = overall mean, $\tau_{\text{ijk}}$ = fixed effect of 8-level treatment factor, $\delta_{l}$ = random effect of block, and $\varepsilon_{\text{ijkl}}$ = residual error. The latter was used to simplify the calculation of the following additional contrasts:

1. Zn level: 30 mg vs. 90 mg
  (PHY+ ZnSO_4_+ and PHY+ ZnO+ vs. PHY+ ZnSO_4_++ and PHY+ ZnO++)
2. Exogenous Zn source: ZnSO_4_ vs. ZnO
  (PHY+ ZnSO_4_+ and PHY+ ZnSO_4_++ vs. PHY+ ZnO+ and PHY+ ZnO++)

Statistical significance was set at *P* < 0.050.

## RESULTS

### Performance Traits

The average body weight of broilers at the start of the experimental phase (d 7) was 153 g. A total of 9 birds (1.4%) died during the experimental phase, but this was not related to treatments. Performance responses were not affected by PHY × Zn source interaction. Phytase supplementation increased ADG by about 6 g/d (*P* < 0.001, Table 3) and reduced FCR by 0.12 (*P* < 0.001). Birds receiving ZnO+ had lower ADFI than birds receiving ZnSO_4_+ or Zn– (*P* = 0.009). Both contrasts (Zn level, exogenous Zn source) were not significant for the performance traits measured.

**Table 3:** Effect of phytase supplementation, Zn source, and dietary level on performance traits in broiler chickens fed the experimental diets from d 7 to d 28.

	Treatment				
Phytase, FTU/kg	Zn source	Zn level, mg/kg	ADG (g/d)	ADFI (g/d)	FCR (g/g)
0	None	0	56.2	85.1	1.52
0	ZnSO_4_	30	56.2	86.3	1.53
0	ZnO	30	56.4	81.7	1.45
750	None	0	63.1	86.5	1.37
750	ZnSO_4_	30	62.5	86.2	1.38
750	ZnO	30	60.7	83.7	1.38
750	ZnSO_4_	90	61.3	86.3	1.41
750	ZnO	90	62.7	86.2	1.38
Pooled SEM			0.98	1.34	0.021
Main effects^1^^,^^2^					
Phytase		0	56.2^b^		1.50^a^
		750	62.1^a^		1.38^b^
Zn source		none		85.8^a^	
		ZnSO_4_		86.3^a^	
		ZnO		82.7^b^	
Pooled SEM			0.59	1.05	0.012
*P*-values					
Zn source			0.503	0.009	0.143
Phytase			<0.001	0.273	<0.001
Zn source × Phytase			0.390	0.693	0.067
Contrast: ZnSO_4_ vs. ZnO			0.842	0.272	0.457
Contrast: 30 mg vs. 90 mg			0.700	0.284	0.576

Abbreviations: ADG, average daily gain; ADFI, average daily feed intake; FCR, feed conversion ratio; SEM, standard error of the mean.

Data are given as least square means; n = 8 pens.

Means within a column not showing a common superscript differ (*P* ≤ 0.050).

1 Main effect estimates represent treatments within the full-factorial design only; treatments with Zn level of 90 mg/kg are not included.

2 Presented if the main effect was significant (*P* ≤ 0.050), and the 2-way interaction was not significant (*P* > 0.050).

### InsP_6_ Disappearance, P, Ca, and Zn Digestibility

The InsP_6_ disappearance, P digestibility and Ca digestibility in duo+jej and by the end of the ileum were unaffected by interaction (PHY × Zn source), Zn source, or level, but were affected by exogenous phytase (Table 4). In PHY– treatments, InsP_6_ disappearance was between 7% and 10.5% (duo+jej) or between 16.5% and 22.1% (ileum). Phytase supplementation increased InsP_6_ disappearance to 51.4% to 54.3% in duo+jej and to 62.8% to 64.7% in the ileum (*P* < 0.001). Digestibility of P and Ca in duo+jej and ileum were increased and decreased, respectively, by phytase supplementation (*P* < 0.001, Table 4). Zinc digestibility in duo+jej and ileum was affected by PHY × Zn source interaction (*P* < 0.010, Table 4). In PHY– treatments, Zn digestibility was at a similar level regardless of Zn source, whereas in PHY+ treatments, Zn digestibility was significantly higher in the Zn– treatment than in the ZnSO_4_+ or ZnO+ treatment. In PHY+ treatments, birds receiving ZnSO_4_ had lower Zn digestibility in duo+jej and ileum than birds receiving ZnO (*P* < 0.050).

**Table 4:** Effect of phytase supplementation (PHY), Zn source and dietary level on Ca, P, and Zn digestibility and InsP_6_ disappearance in duodenum and jejunum (duo + jej) and ileum in broiler chickens fed the experimental diets from d 7 to d 28.

Treatment			InsP_6_ disappearance		P digestibility		Ca digestibility		Zn digestibility	
			%		%		%		%	
			Duo+Jej	Ileum	Duo+Jej	Ileum	Duo+Jej	Ileum	Duo+Jej	Ileum
Phytase, FTU/kg	Zn source	Zn level								

0	None	0	9.9	21.1	27.5	40.2	37.1	50.8	6.0^b^	16.8^b^
0	ZnSO_4_	30	10.5	22.1	28.3	40.7	36.5	51.6	5.2^b^	15.4^b^
0	ZnO	30	7.0	16.5	28.1	39.6	37.5	51.6	3.6^b^	12.5^bcd^
750	None	0	54.3	62.8	41.5	53.2	29.4	39.5	13.0^a^	24.3^a^
750	ZnSO_4_	30	53.9	64.3	43.3	55.3	29.4	40.8	-2.5^c^	9.7^cd^
750	ZnO	30	51.6	64.7	43.1	55.4	30.7	43.0	3.1^bc^	14.0^bc^
750	ZnSO_4_	90	52.4	66.3	42.8	56.2	29.5	42.5	0.1^bc^	8.0^d^
750	ZnO	90	51.4	63.8	40.9	53.7	27.8	39.9	4.8^b^	12.8^bcd^
Pooled SEM			1.91	1.87	1.15	1.18	0.99	1.38	2.05	1.92
Main effects^1^^,^^2^										
Phytase		0	9.1^b^	19.9^b^	28.0^b^	40.2^b^	37.0^a^	51.3^a^		
		750	53.3^a^	63.9^a^	42.6^a^	54.6^a^	29.8^b^	41.1^b^		
Pooled SEM			1.15	1.13	0.74	0.89	0.65	1.14		
Contrasts										
Exogenous Zn source										
		ZnSO_4_							−1.2^b^	8.9^b^
		ZnO							4.0^a^	13.4^a^
Pooled SEM									2.05	1.92
*P*-values										
Zn source			0.213	0.365	0.430	0.402	0.397	0.085	<0.001	<0.001
Phytase			<0.001	<0.001	<0.001	<0.001	<0.001	<0.001	0.818	0.495
Zn source × Phytase			0.944	0.153	0.878	0.336	0.875	0.327	0.003	0.005
Contrast: ZnSO_4_ vs. ZnO			0.381	0.553	0.320	0.216	0.876	0.832	0.016	0.021
Contrast: 30mg vs. 90 mg			0.651	0.774	0.213	0.720	0.128	0.491	0.293	0.456

SEM, standard error of the mean. Data are given as least square means; n = 8 pens.

Means within a column not showing a common superscript differ (P ≤ 0.050).

1 Main effect estimates represent treatments within the full-factorial design only; treatments with Zn level of 90 mg/kg are not included.

2 Presented if the main effect was significant (*P* ≤ 0.050), and the 2-way interaction was not significant (*P* > 0.050).

In duo+jej, concentrations of InsP_6_ and Ins(1,2,4,5,6)P_5_ were not affected by interaction (PHY × Zn source), Zn source, or level but by exogenous phytase (Table 5). Birds fed PHY+ treatments had lower concentrations of InsP_6_ (*P* < 0.001) and higher concentrations of Ins(1,2,4,5,6)P_5_ (*P* = 0.003) than birds fed PHY– treatments. Birds fed PHY– treatments had similar Ins(1,2,3,4,5)P_5_ concentrations (1.3–1.7 μmol/g DM) regardless of Zn source; whereas, in PHY+ treatments, birds fed Zn– had lower Ins(1,2,3,4,5)P_5_ concentrations than birds fed ZnO+ (PHY × Zn source: *P* = 0.008). In PHY+ treatments, 30 mg Zn supplementation tended (*P* = 0.095) to result in higher Ins(1,2,3,4,5)P_5_ concentrations than supplementation with 90 mg Zn. In PHY– treatments, the concentration of Ins(1,2,3,4)P_4_ was similar when Zn–, ZnSO_4_+ or ZnO+ was fed, but in PHY+ treatments, birds receiving Zn– showed lower concentrations of this isomer than birds receiving Zn supplementation (PHY × Zn source: *P* = 0.008). The isomers Ins(1,2,5,6)P_4_ and InsP_3x_ were only detectable in PHY+ treatments, whereby only Ins(1,2,5,6)P_4_ occurred at a quantifiable level.

**Table 5:** Effect of phytase supplementation (PHY), Zn source and dietary level on InsP_x_ concentrations (μmol/g DM) in duodenum and jejunum (duo + jej) in broiler chickens fed the experimental diets from d 7 to d 28.

	Treatment								
Phytase, FTU/kg	Zn source	Zn level, mg/kg	InsP_6_	Ins(1,2,4,5,6)P_5_	Ins(1,2,3,4,5)P_5_	Ins(1,2,3,4,6)P_5_	Ins(1,2,5,6)P_4_	Ins(1,2,3,4)P_4_	InsP_3x_^1^
0	None	0	31.2	1.2	1.7^c^	0.7	<LOQ	0.4^d^	n.d.^2^
0	ZnSO_4_	30	31.6	1.2	1.6^c^	0.7	<LOQ	0.5^d^	n.d.
0	ZnO	30	31.2	1.2	1.3^c^	0.6	<LOQ	0.4^d^	n.d.
750	None	0	15.0	1.3	3.3^b^	<LOQ^3^	4.4	0.6^c^	1.8
750	ZnSO_4_	30	15.9	1.3	3.5^ab^	<LOQ	3.8	0.7^b^	1.8
750	ZnO	30	17.1	1.5	3.9^a^	<LOQ	4.1	0.8^ab^	1.8
750	ZnSO_4_	90	16.6	1.4	3.6^ab^	<LOQ	3.7	0.8^a^	1.7
750	ZnO	90	16.3	1.3	3.3^b^	<LOQ	3.5	0.7^ab^	1.7
Pooled SEM			0.71	0.07	0.14	.	.	0.03	.
Main effects^4^^,^^5^									
Phytase		0	31.3^a^	1.2^b^					
		750	16.0^b^	1.3^a^					
Pooled SEM			0.47	0.05					
*P*-values									
Zn source			0.274	0.261	0.668	.	.	0.016	.
Phytase			<0.001	0.003	<0.001	.	.	<0.001	.
Zn source × Phytase			0.255	0.200	0.008	.	.	0.008	.
Contrast: ZnSO_4_ vs. ZnO			0.507	0.568	0.722	.	.	0.520	.
Contrast: 30 mg vs. 90 mg			0.970	0.306	0.095	.	.	0.285	.

SEM, standard error of the mean. Data are given as least square means; n = 8 pens.

Means within a column not showing a common superscript differ (*P* ≤ 0.050).

1 At least one of the following isomers: Ins(1,2,6)P_3_, Ins(1,4,5)P_3_, Ins(2,4,5)P_3_.

2 n.d.: not detectable in majority of samples, limit of detection of InsP_3x_: 0.3 μmol/g.

3 <LOQ: not quantifiable in majority of samples, limit of quantification of Ins(1,2,3,4,6)P_5_ and Ins(1,2,5,6)P_4_: 0.3 μmol/g.

4 Main effect estimates represent treatments within the full-factorial design only; treatments with Zn level of 90 mg/kg are not included.

5 Presented if the main effect was significant (*P* ≤ 0.050), and the 2-way interaction was not significant (*P* > 0.050).

Ileal concentrations of InsP_3-6_ were not affected by interaction (PHY × Zn source), Zn source, or level, but only by exogenous phytase (*P* < 0.001, Table 6).

**Table 6:** Effect of phytase supplementation (PHY), Zn source, and dietary level on InsP_x_ concentrations (μmol/g DM) in ileum in broiler chickens fed the experimental diets from d 7 to d 28.

Treatment										
Phytase, FTU/kg	Zn source	Zn level, mg/kg		InsP_6_	Ins(1,2,4,5,6)P_5_	Ins(1,2,3,4,5)P_5_	Ins(1,2,3,4,6)P_5_	Ins(1,2,5,6)P_4_	Ins(1,2,3,4)P_4_	InsP_3x_^1^
0	None	0	46.5		1.8	2.4	1.1	<LOQ	0.5	<LOQ
0	ZnSO_4_	30	46.5		1.8	2.3	1.1	<LOQ	0.6	<LOQ
0	ZnO	30	45.6		1.7	2.1	1.1	<LOQ	0.7	<LOQ
750	None	0	20.5		2.1	5.5	<LOQ^2^	9.2	1.0	3.1
750	ZnSO_4_	30	20.2		2.1	5.3	<LOQ	7.3	1.0	2.9
750	ZnO	30	19.9		2.0	5.4	<LOQ	7.7	1.0	3.2
750	ZnSO_4_	90	18.9		1.9	5.1	<LOQ	6.9	1.1	3.1
750	ZnO	90	20.3		1.9	5.0	<LOQ	6.6	1.1	3.2
Pooled SEM			1.18		0.11	0.27	.	.	0.05	.
Main effects^3^^,^^4^										
Phytase		0	46.2^a^		1.8^b^	2.3^b^			0.6^b^	
		750	20.2^b^		2.1^a^	5.4^a^			1.0^a^	
Pooled SEM			0.78		0.08	0.17	.	.	0.03	.
*P*-values										
Zn source			0.788		0.734	0.597	.	.	0.173	.
Phytase			<0.001		<0.001	<0.001	.	.	<0.001	.
Zn source × Phytase			0.970		0.957	0.817	.	.	0.340	.
Contrast: ZnSO_4_ vs. ZnO			0.598		0.888	0.800	.	.	0.636	.
Contrast: 30 mg vs. 90 mg			0.697		0.262	0.247	.	.	0.479	.

SEM, standard error of the mean. Data are given as least square means; n = 8 pens.

Means within a column not showing a common superscript differ (*P* ≤ 0.050).

1 At least one of the following isomers: Ins(1,2,6)P_3_, Ins(1,4,5)P_3_, Ins(2,4,5)P_3_.

2 <LOQ: not quantifiable in majority of samples, limit of quantification of Ins(1,2,3,4,6)P_5_ and Ins(1,2,5,6)P_4_: 0.3 μmol/g.

3 Main effect estimates represent treatments within the full-factorial design only; treatments with Zn level of 90 mg/kg are not included.

4 Presented if the main effect was significant (*P* ≤ 0.050), and the 2-way interaction was not significant (*P* > 0.050).

### *Myo*-Inositol

*Myo*-inositol concentration in duo+jej was increased in PHY+ treatments (Table 7; *P* < 0.001). Ileal MI concentration was affected by PHY × Zn source interaction (*P* = 0.030). The lowest (*P* < 0.050) ileal MI concentration was observed in broilers fed PHY– treatments. The ileal MI concentration observed in birds fed PHY+ZnSO_4_+ or PHY+ZnO+ was greater than in birds fed the PHY+Zn– treatment (*P* < 0.050). In plasma, the MI concentration was increased by phytase supplementation (*P* < 0.001) but was unaffected by Zn source. Birds fed 30 mg/kg Zn and phytase supplementation had higher plasma MI concentrations than birds fed 90 mg Zn and phytase supplementation (*P* = 0.013).

**Table 7:** Effect of phytase supplementation (PHY), Zn source, and dietary level on *myo*-inositol concentration in duodenum and jejunum (duo + jej), ileum and plasma of broiler chickens fed the experimental diets from d 7 to d 28.

				*Myo*-inositol		
				Duo+jej	Ileum	Plasma
Treatment				μmol/g DM	μmol/g DM	μmol/ml
Phytase, FTU/kg	Zn source		Zn level, mg/kg			

0	None		0	6.3	1.8^c^	0.24
0	ZnSO_4_		30	6.7	1.8^c^	0.24
0	ZnO		30	5.7	2.1^c^	0.21
750	None		0	11.7	4.7^b^	0.39
750	ZnSO_4_		30	12.2	5.9^a^	0.37
750	ZnO		30	12.5	6.7^a^	0.38
750	ZnSO_4_		90	11.5	6.1^a^	0.30
750	ZnO		90	12.3	5.9^a^	0.35
Pooled SEM				0.56	0.40	0.019
Main effects^1^^,^^2^						
Phytase	0			6.2^b^		0.23^b^
	750			12.1^a^		0.38^a^
Pooled SEM				0.38		0.012
Contrasts						
Zn level	30					0.38^a^
	90					0.33^b^
Pooled SEM						0.018
*P*-values						
Zn source				0.648	0.005	0.568
Phytase				<0.001	<0.001	<0.001
Zn source × Phytase				0.327	0.030	0.509
Contrast: ZnSO_4_ vs. ZnO				0.288	0.378	0.098
Contrast: 30 mg vs. 90 mg				0.412	0.359	0.013

SEM, standard error of the mean. Data are given as least square means; n = 8 pens.

Means within a column not showing a common superscript differ (*P* ≤ 0.050).

1 Main effect estimates represent treatments within the full-factorial design only; treatments with Zn level of 90 mg/kg are not included.

2 Presented if the main effect was significant (*P* ≤ 0.050), and the 2-way interaction was not significant (*P* > 0.050).

### Blood Minerals and Liver

A significant interaction (PHY × Zn source) was found for serum Zn concentrations (*P* = 0.014, Table 8). Lowest Zn concentrations were observed for the serum of broilers fed the PHY–Zn– treatment. Adding either an exogenous Zn source, phytase, or both, increased serum Zn concentrations (*P* < 0.001). Phytase supplementation increased serum P_i_ concentrations and decreased serum Ca concentrations (*P* < 0.001). Liver Zn concentrations were higher for birds receiving Zn supplemented treatments independent of exogenous Zn source (*P* = 0.004). Phytase supplementation increased liver Zn concentration, total liver Zn quantity, and liver weight (*P* < 0.050). However, the phytase effect on liver weight disappeared when liver weights were corrected for the individual body weight of sampled animals (*P* > 0.050).

**Table 8:** Effect of phytase supplementation (PHY), Zn source, and dietary level on blood minerals and liver characteristics in broiler chickens fed the experimental diets from d 7 to d 28.

			Serum			Liver			
			Zn conc	P_i_ conc	Ca conc	Zn conc	Zn	Weight	Weight^1^
Treatment			μg/L	mmol/L	mmol/L	mg/kg DM	mg	g	g/g
Phytase, FTU/kg	Zn source	Zn level, mg/kg							

0	None	0	712^d^	2.0	2.9	68.1	2.2	32.1	0.024
0	ZnSO_4_	30	1,264^bc^	2.1	2.9	71.7	2.4	32.7	0.025
0	ZnO	30	1,338^abc^	2.1	3.0	77.0	2.3	30.1	0.023
750	None	0	1,207^c^	2.9	2.7	72.0	2.7	37.7	0.024
750	ZnSO_4_	30	1,435^ab^	2.8	2.6	78.6	3.0	38.3	0.025
750	ZnO	30	1,404^abc^	2.8	2.6	75.5	2.5	33.9	0.024
750	ZnSO_4_	90	1,383^abc^	2.9	2.6	75.3	2.5	33.7	0.023
750	ZnO	90	1,543^a^	2.9	2.7	77.5	2.8	35.9	0.024
Pooled SEM			73.92	0.08	0.06	2.08	0.18	2.53	0.0010
Main effects^2^^,^^3^									
Phytase		0		2.1^b^	2.9^a^	72.2^b^	2.3^b^	31.7^b^	
		750		2.8^a^	2.6^b^	75.3^a^	2.7^a^	36.6^a^	
Pooled SEM				0.05	0.04	1.41	0.10	1.46	
Zn source		None				70.0^b^			
		ZnSO_4_				75.1^a^			
		ZnO				76.2^a^			
Pooled SEM						1.61			
*P*-values									
Zn source			<0.001	0.698	0.743	0.004	0.307	0.341	0.236
Phytase			<0.001	<0.001	<0.001	0.048	0.002	0.019	0.435
Zn source × Phytase			0.014	0.547	0.232	0.088	0.475	0.919	0.810
Contrast: ZnSO_4_ vs. ZnO			0.382	0.934	0.543	0.801	0.552	0.656	0.784
Contrast: 30 mg vs. 90 mg			0.557	0.219	0.543	0.735	0.599	0.609	0.175

SEM; standard error of the mean. Data are given as least square means; n = 8 pens.

Means within a column not showing a common superscript differ (*P* ≤ 0.050).

P_i_: inorganic P.

1 Liver weight corrected for individual body weight.

2 Main effect estimates represent treatments within the full-factorial design only; treatments with Zn level of 90 mg/kg are not included.

3 Presented if the main effect was significant (*P* ≤ 0.050), and the 2-way interaction was not significant (*P* > 0.050).

### Bone Ash

Tibia and foot ash concentrations were increased by exogenous phytase (*P* < 0.001, Tables 9 and 10). Zinc concentrations in the tibia and foot ash were affected by interaction of PHY × Zn source (*P* = 0.006). Both Zn and phytase supplementation significantly increased Zn concentrations in the tibia and foot ash, but the effect of Zn supplementation was greater than the effect of phytase supplementation. Birds receiving 30 mg Zn tended to have lower Zn concentrations in the tibia ash and foot ash than birds receiving 90 mg Zn (*P* < 0.100). Phytase supplementation decreased Ca concentrations in tibia ash (*P* = 0.003), and increased Ca concentrations in foot ash (*P* = 0.010). The concentration of P in tibia and foot ash was higher in PHY+ treatments compared to PHY– treatments (*P* < 0.001).

**Table 9:** Effect of phytase supplementation (PHY), Zn source, and dietary level on tibia mineralization in broiler chickens fed the experimental diets from d 7 to d 28.

						Minerals in tibia ash					
				Tibia ash^1^		Zn	Ca	P	Zn	Ca	P
Treatment				%	g/bone	mg/kg	g/kg	g/kg	mg	g	g
Phytase, FTU/kg	Zn source		Zn level, mg/kg								

0	None		0	40.5	1.24	204^d^	362	179	0.25	0.45	0.22
0	ZnSO_4_		30	41.3	1.25	329^b^	363	180	0.41	0.45	0.23
0	ZnO		30	42.2	1.29	341^b^	365	180	0.44	0.47	0.23
750	None		0	46.0	1.67	298^c^	360	183	0.50	0.60	0.31
750	ZnSO_4_		30	46.1	1.66	378^a^	361	182	0.63	0.60	0.31
750	ZnO		30	45.3	1.57	389^a^	362	183	0.61	0.57	0.29
750	ZnSO_4_		90	45.8	1.64	398^a^	359	182	0.65	0.59	0.30
750	ZnO		90	46.0	1.61	397^a^	362	183	0.64	0.58	0.30
Pooled SEM				0.49	0.053	8.10	3.03	1.35	0.022	0.021	0.010
Main effects^2^^,^^3^											
Phytase			0	41.3^b^	1.26^b^		363.4^a^	179.5^b^	0.37^b^	0.46^b^	0.23^b^
			750	45.8^a^	1.63^a^		360.5^b^	182.9^a^	0.58^a^	0.59^a^	0.30^a^
Pooled SEM				0.28	0.032		2.89	1.26	0.013	0.015	0.007
Zn source			none						0.37^b^		
			ZnSO_4_						0.52^a^		
			ZnO						0.52^a^		
Pooled SEM									0.016		
*P*-values											
Zn source				0.591	0.793	<0.001	0.113	0.668	<0.001	0.891	0.771
Phytase				<0.001	<0.001	<0.001	0.003	<0.001	<0.001	<0.001	<0.001
Zn source × Phytase				0.054	0.276	0.006	0.889	0.068	0.233	0.310	0.278
Contrast: ZnSO_4_ vs. ZnO				0.572	0.210	0.494	0.125	0.185	0.414	0.262	0.270
Contrast: 30 mg vs. 90 mg				0.644	0.848	0.084	0.657	0.357	0.227	0.894	0.948

SEM, standard error of the mean. Data are given as least square means; n = 8 pens.

Means within a column not showing a common superscript differ (*P* ≤ 0.050).

1 Dry matter basis.

2 Main effect estimates represent treatments within the full-factorial design only; treatments with Zn level of 90 mg/kg are not included.

3 Presented if the main effect was significant (*P* ≤ 0.050), and the 2-way interaction was not significant (*P* > 0.050).

**Table 10:** Effect of phytase supplementation (PHY), Zn source, and dietary level on foot mineralization in broiler chickens fed the experimental diet from d 7 to d 28.

					Minerals in foot ash					
			Foot ash^1^		Zn	Ca	P	Zn	Ca	P
Treatment			%	g/bone	mg/kg	g/kg	g/kg	mg	g	g
Phytase, FTU/kg	Zn source	Zn level, mg/kg								

0	None	0	13.5	1.36	249^d^	337	175	0.34	0.46	0.24
0	ZnSO_4_	30	13.8	1.37	355^b^	336	176	0.49	0.46	0.24
0	ZnO	30	14.2	1.39	368^b^	340	176	0.51	0.47	0.25
750	None	0	15.7	1.74	325^c^	341	181	0.57	0.60	0.32
750	ZnSO_4_	30	15.9	1.77	395^a^	342	181	0.70	0.60	0.32
750	ZnO	30	15.8	1.67	398^a^	343	182	0.67	0.57	0.30
750	ZnSO_4_	90	15.8	1.70	415^a^	342	182	0.70	0.58	0.31
750	ZnO	90	15.5	1.67	407^a^	341	181	0.68	0.57	0.30
Pooled SEM			0.23	0.042	7.98	2.13	1.08	0.018	0.015	0.008
Main effects^2^^,^^3^										
Phytase		0	13.8^b^	1.37^b^		338^b^	176^b^	0.45^b^	0.46^b^	0.24^b^
		750	15.8^a^	1.73^a^		342^a^	182^a^	0.64^a^	0.59^a^	0.31^a^
Pooled SEM			0.13	0.027		1.41	0.70	0.010	0.010	0.005
Zn source		none						0.45^b^		
		ZnSO_4_						0.59^a^		
		ZnO						0.59^a^		
Pooled SEM								0.013		
*P*-values										
Zn source			0.248	0.647	<0.001	0.283	0.649	<0.001	0.813	0.710
Phytase			<0.001	<0.001	<0.001	0.010	<0.001	<0.001	<0.001	<0.001
Zn source × Phytase			0.306	0.271	0.006	0.838	0.794	0.093	0.248	0.155
Contrast: ZnSO_4_ vs. ZnO			0.424	0.115	0.746	1.000	0.902	0.111	0.143	0.103
Contrast: 30 mg vs. 90 mg			0.424	0.364	0.056	0.611	0.539	0.608	0.340	0.410

SEM, standard error of the mean. Data are given as least square means; n = 8 pens.

Means within a column not showing a common superscript differ (*P* ≤ 0.050).

1 Dry matter basis.

2 Main effect estimates represent treatments within the full-factorial design only; treatments with Zn level of 90 mg/kg are not included.

3 Presented if the main effect was significant (*P* ≤ 0.050), and the 2-way interaction was not significant (*P* > 0.050).

## DISCUSSION

The first hypothesis that Zn supplementation reduces the efficacy of exogenous phytase at a dose of 750 FTU/kg could not be confirmed as the prececal InsP_6_ disappearance, the prececal P digestibility and the bone ash concentrations were not affected by dietary Zn level or Zn source (solubility). In contrast, the second hypothesis, that phytase supplementation increases the Zn concentrations in bone, blood, and liver was confirmed.

### Effects of Zn Source and Level on Phytase Efficacy

The supplementation of phytase significantly increased the InsP_6_ disappearance in duo+jej and by the end of the ileum, and the concentrations of lower InsP_x_ in duo+jej and ileum, confirming results from previous studies (Zeller et al., 2015; Sommerfeld et al., 2018b; Künzel et al., 2021). An interaction of phytase and Zn source was only observed in duo+jej for Ins(1,2,3,4)P_4_ and Ins(1,2,3,4,5)P_5_. The concentration of both isomers was not affected by the Zn source in PHY– treatments but in PHY+ treatments. However, this Zn source effect on some lower InsP_x_ was not significant anymore by the end of the ileum. The absence of interaction (PHY × Zn source) and the non-significant contrast for 30 mg vs. 90 mg Zn supplementation for InsP_6_ disappearance and accumulation of lower InsP_x_ by the end of the ileum showed that neither Zn source nor Zn level affected phytase efficacy on a level that is relevant for the bird. Of note, the present study used an InsP_6_-P concentration in the feed of 3.3 g/kg DM, which was higher than in common diets based on soybean meal, corn, and other cereal grains. This was intended to provide a high amount of substrate for Zn to bind to and the exogenous enzyme to hydrolyze, thus creating a greater potential for interactions in the digestive tract.

Changes in phytase efficacy could occur through interactions between Zn and phytate. The results of the present study confirm the hypothesis of Schlegel et al. (2010) that supplemented Zn, independent of the source (chelates or sulfates), does not interact with phytate, at least not to the extent that it would influence InsP_6_ degradation. However, this finding contradicts in vitro studies that reported a negative effect of Zn on phytase efficacy (Maenz et al., 1999), which depends on Zn level and source (Santos et al., 2015). Differences in the results of in vitro studies compared to the present study might be attributed to the different sources of InsP_6_, which can impact phytase efficacy and susceptibility to interaction with minerals (Sommerfeld and Santos, 2021). In vitro studies often use Na phytate (Sommerfeld and Santos, 2021), whereas in the present study, InsP_6_ originated from corn, soybean meal, rapeseed meal, and rice bran. Also, different phytase origins (e.g., *E. coli* or *Aspergillus niger*) might cause contradictory results, as they seem to have different sensitivity to Zn supplementation (Santos et al., 2015). Another difference between in vitro studies (Santos et al., 2015) and the present in vivo study is the order in which the reactions or interactions between Zn and phytate or exogenous phytase and phytate occur. In vitro, all interactions occur simultaneously, while in vivo, different conditions along the digestive tract cause potential interactions to occur in different sections to a different degree. Further, the lack of interaction of PHY × Zn source contradicts an in vivo study, where adverse effects of Zn supplementation on tibia ash were found in the presence of exogenous phytase (Augspurger et al., 2004). The conflicting results might be attributed to the different Zn levels. In the study by Augspurger et al. (2004), the Zn level was much higher (800 mg/kg, Zn from Zn chloride) than the highest Zn level used in the present study. Zinc digestibility data on an intestinal level could not provide further insight into interactions of Zn and phytate. Because of endogenous Zn losses that depend on the level of Zn supply (Brugger and Windisch, 2019), the value of Zn digestibility data to study Zn interactions is limited.

*Myo*-inositol is obtained by complete dephosphorylation of InsP_6_ in the gastrointestinal tract. To our knowledge, this is the first study looking at intestinal MI concentrations concerning dietary Zn level and Zn source in broiler chickens. As observed in other studies, a consistent increase of MI concentration was found in duo+jej, ileum, and plasma due to phytase supplementation (Beeson et al., 2017; Sommerfeld et al., 2018a,b; Novotny et al., 2023). The supplementation of Zn, in addition to exogenous phytase, resulted in increased ileal MI concentrations compared to phytase supplementation alone. The lower ileal MI concentration in the PHY+ Zn– treatment is consistent with the numerically higher ileal concentration of the isomers Ins(1,2,5,6)P_4_ of that treatment compared to the PHY+ ZnSO_4_+ and PHY+ ZnO+ treatments, indicating a decelerated degradation and leading to lower ileal MI concentrations in the PHY+ Zn– treatment. As Zn is a cofactor for alkaline phosphatase (McCall et al., 2000), Zn supplementation might increase endogenous alkaline phosphatase activity (Williams, 1972). It can be assumed that 2 different “types” of interaction can affect the activity of alkaline phosphatases. On the one hand, in treatments without Zn supplementation, an internal interaction (triggered by Zn deficiency) could influence the activity of the phosphatases. On the other hand, an external interaction between Zn and phytate/phytase could influence the activity. Endogenous phosphatases are produced by either enterocytes or microorganisms and contribute to the degradation of InsP, especially of lower InsP (Huber, 2016; Rodehutscord and Rosenfelder, 2016). However, no differences in the ileal MI concentrations were observed due to Zn in PHY– treatments. The lack of an effect of Zn in PHY– treatments could be due to a lower mucosal phosphatase activity due to the reduced intestinal flow of lower InsPs in PHY– treatments compared with PHY+ treatments (Novotny et al., 2023). The smaller scope for Zn to affect the activity of phosphatases in PHY– treatments might have caused that the effect of Zn was not visible.

As expected, phytase supplementation significantly increased P digestibility in duo+jej and by the end of the ileum, and bone ash. Although it has been suggested for piglets that Ca^2+^ and Zn^2+^ compete for a non-specific transporter in the brush border membrane (Bertolo et al., 2001), in the present study, no effects of Zn source or dietary Zn level were found on Ca digestibility. However, phytase supplementation significantly decreased Ca digestibility in duo+jej and by the end of the ileum. Previously published studies on this topic show divergent results. Some studies observed an increase in prececal Ca digestibility due to phytase supplementation (Sommerfeld et al., 2018a; Krieg et al., 2021; Li et al., 2021), while others found no effect (Sommerfeld et al., 2018b; Krieg et al., 2021) or a decreased prececal Ca digestibility (Olukosi et al., 2013; Krieg et al., 2021). In contrast to P, where excretion with urine is low, Ca excretion with urine is variable and can be markedly high. Thus, complementary collection of total excreta is needed for a comprehensive evaluation of effects on Ca metabolism.

As shown in several other studies (Künzel et al., 2019; Babatunde et al., 2020; Künzel et al., 2021), tibia and foot ash quantity (g/bone), as well as tibia and foot ash concentration were significantly increased by phytase supplementation. Consistent with that, P and Ca quantities and P concentration in tibia and foot ash were significantly increased with phytase supplementation. However, Ca concentration in tibia ash was significantly reduced due to phytase supplementation. The response to phytase supplementation in terms of P concentrations showed similar pattern in foot ash and blood serum (coefficient of correlation (r) = 0.73, *P* < 0.001). Phytase supplementation increased P_i_ serum concentration and decreased Ca serum concentration, which has also been observed in other studies (Sebastian et al., 1996; Künzel et al., 2019). The homeostasis of Ca and P is interdependent. Low P supply of birds receiving PHY– treatments might have caused a reduced incorporation of P and Ca into the bones to maintain the required blood P level, leading to elevated blood Ca levels (Proszkowiec-Weglarz and Angel, 2013).

The present study confirmed results from Schlegel et al. (2010) and Olukosi et al. (2018) that tibia ash concentrations were not affected by dietary Zn level or Zn source. In contrast, Ao et al. (2007) found an increase in tibia ash (mg/tibia) due to the supplementation of a chelated Zn source, and Augspurger et al. (2004) observed a decrease in tibia ash concentration and quantity in treatments supplemented with phytase and high levels of Zn chloride (800 mg/kg Zn). As mentioned before, differences between the latter and the present study might be partly explained by the different Zn supplementation levels. Another factor might be the exogenous Zn source used. The 2 exogenous Zn sources used in the present study showed no difference in their effect on phytase efficacy or related measures. However, a difference between other Zn sources than ZnO (HiZox) and ZnSO_4_ cannot be ruled out.

### Effects of Phytase Supplementation on Zn Traits

All Zn traits investigated were highly correlated with one another, particularly bone and serum Zn concentrations (Zn tibia ash and Zn foot ash concentration: r = 0.98; Zn serum and Zn tibia ash concentration: r = 0.78; (*P* < 0.001)). As observed in other studies, phytase supplementation increased Zn concentrations in serum and tibia ash (Ao et al., 2007; Schlegel et al., 2010). This increase can be attributed to the increased degradation of InsP_6_ in the gastrointestinal tract and the associated release of Zn (Yi et al., 1996; Mohanna and Nys, 1999a). In the present study, 30 mg/kg Zn supplementation further increased Zn concentrations in serum and tibia ash in PHY+ treatments, whereby the effect was independent of the exogenous Zn source used. A supplementation of 90 mg/kg Zn did not further increase Zn concentrations in serum and tibia ash. No difference due to the exogenous Zn source (15 mg/kg Zn as ZnSO_4_ vs. Zn glycine chelate) was also observed in the study by Schlegel et al. (2010). However, unlike the present study, combining phytase and Zn supplementation did not further increase Zn concentrations in plasma. Previous studies have reported that blood and tibia Zn concentrations plateau at a certain level of Zn supplementation (Wedekind et al., 1992; Mohanna and Nys, 1999b). The higher dietary phytate-P concentration and the lower dietary intrinsic Zn concentration in the present study possibly caused the blood Zn concentration to plateau only when Zn and phytase were supplemented together. In contrast, in the study of Schlegel et al. (2010) a plateau of blood Zn concentration was probably already reached with phytase supplementation alone, whereas the tibia ash Zn concentration did not reach a plateau with phytase supplementation alone. As in the present study, Ao et al. (2007) observed increased liver Zn concentrations due to Zn supplementation. However, contrary to the present study, phytase supplementation in their study did not significantly affect liver Zn concentration. The difference might be partly explained by the different phytase supplementation levels (750 FTU/kg vs. 500 FTU/kg) and dietary phytate-P concentrations. In the present study, no differences in liver Zn concentrations were found depending on the exogenous Zn source used. Consistent with this, no differences in liver Zn concentrations were observed by Azad et al. (2017) when comparing ZnSO_4_ with Zn methionine and Zn-enriched yeast.

With respect to the Zn status, growth performance appears not to be a sensitive trait in the present study. The ADG was not affected by Zn source or supplementation level indicating that intrinsic Zn was sufficient for growth when phytase was added. This is consistent with the results from studies by Schlegel et al. (2010) and Burrell et al. (2004), whereas in other studies the intrinsic Zn concentration was not sufficient for growth (Yi et al., 1996; Ao et al., 2007). Compared to the present study, intrinsic Zn concentrations were lower in the studies where Zn was limiting for growth (30 mg/kg vs. 20 mg/kg, 25 mg/kg). As mentioned before, the diet composition affects the availability of intrinsic Zn, which makes it difficult to determine the optimal Zn content in broiler diets.

## Conclusion and Implications

In conclusion, Zn supplementation at nonpharmacologic levels did not reduce exogenous phytase efficacy. Previously reported effects of Zn supplementation on phytase efficacy could be due to very high Zn supplementation levels or differences in the solubility of InsP_6_ sources. Further studies are required to rule out an effect on exogenous phytase efficacy from Zn sources other than those tested in this study. Also, further research is needed on the activity of endogenous phosphatases, as the effect of Zn supplementation on these enzymes requires further investigation. It was confirmed that phytase supplementation increases the Zn concentrations in bone, blood serum, and liver. Since in the presence of phytase, supplementation of 90 mg/kg Zn did not confer any benefit in comparison to 30 mg/kg Zn, the level of Zn supplementation in phytase-supplemented broiler diets used by the industry may be reconsidered.
